# Action Video Games Enhance Attentional Control and Phonological Decoding in Children with Developmental Dyslexia

**DOI:** 10.3390/brainsci11020171

**Published:** 2021-01-29

**Authors:** Sara Bertoni, Sandro Franceschini, Giovanna Puccio, Martina Mancarella, Simone Gori, Andrea Facoetti

**Affiliations:** 1 Department of Human and Social Sciences, University of Bergamo, 24129 Bergamo, Italy; simone.gori@unibg.it; 2 Department of General Psychology, University of Padova, 35122 Padova, Italy; sandro.franceschini@unipd.it (S.F.); giovanna.puccio@studenti.unipd.it (G.P.); martina.mancarella@kuleuven.be (M.M.); andreafacoetti@unipd.it (A.F.); 3 Katholieke Universiteit Leuven, 3000 Leuven, Belgium

**Keywords:** visual spatial attention, attentional training, reading disorder, sub-lexical route, phonological dyslexia, executive functions, top-down control, prefrontal cortex, goal-directed attention, frontal eye fields, posterior parietal cortex, stimulus-driven attention, magnocellular-dorsal pathway

## Abstract

Reading acquisition is extremely difficult for about 5% of children because they are affected by a heritable neurobiological disorder called developmental dyslexia (DD). Intervention studies can be used to investigate the causal role of neurocognitive deficits in DD. Recently, it has been proposed that action video games (AVGs)—enhancing attentional control—could improve perception and working memory as well as reading skills. In a partial crossover intervention study, we investigated the effect of AVG and non-AVG training on attentional control using a conjunction visual search task in children with DD. We also measured the non-alphanumeric rapid automatized naming (RAN), phonological decoding and word reading before and after AVG and non-AVG training. After both video game training sessions no effect was found in non-alphanumeric RAN and in word reading performance. However, after only 12 h of AVG training the attentional control was improved (i.e., the set-size slopes were flatter in visual search) and phonological decoding speed was accelerated. Crucially, attentional control and phonological decoding speed were increased only in DD children whose video game score was highly efficient after the AVG training. We demonstrated that only an efficient AVG training induces a plasticity of the fronto-parietal attentional control linked to a selective phonological decoding improvement in children with DD.

## 1. Introduction

One of the most important cognitive skills in modern society is reading, which starts its development with formal school education. However, for about 5% of children learning to read is extremely difficult because they are affected by a heritable neurobiological disorder called developmental dyslexia (DD). Reading performance of children with DD is often characterized by the presence of multiple errors, and, especially in shallow orthographies, reading is extremely slow. Moreover, during the years of education reading speed shows a reduced growth trend compared to typical readers [1].

There are several theories and different approaches to study and treat DD. The most popular one is the phonological theory, in which the core deficit of DD is identified in an auditory and phonological processing impairment [2,3]. Based on this theory, some intervention programs for DD are focused on improving phonological processing [4], but the improvements are often found in reading accuracy of single word and pseudoword and in letter-sound knowledge, rather than in text reading fluency (i.e., the ability to read text and pseudowords rapidly and accurately; [5]). For these reasons the intervention programs based only on phonological domains could be not so effective [6].

DD has been studied considering other deficits as possible causes, and DD is now considered a multifactorial neurodevelopmental disorder, with multiple causes co-occurring in this complex framework [7]. Indeed, several cognitive skills during the pre-reading stage lay the groundwork for later reading development, and these cognitive skills are often impaired in children with DD [8,9,10,11].

In particular, there are general-domain cognitive skills, such as visuo-spatial attention, that are involved in the reading processes [12,13,14,15,16,17]. Spatial attention allows to allocate selective attention enhancing specific processing for target objects at particular locations within the visual field [18]. Selective attention, reducing the impact of irrelevant information, is fundamental to allowing task-relevant information to guide perception and other cognitive domains such as memory and decision making [15,18]. Letter identification is a fundamental stage in phonological decoding, visual word recognition and contextual reading fluency [19,20,21], and the endogenous control of top-down attention is used to move the spatial attention rapidly over the targeted letter string. Thus, a general impairment in selective spatial attention could reduce the efficiency in filtering irrelevant or distractors information, such as the letters in a word or a word in a text that surround the target letter or word [8,9,10,12,13,14]. The efficient orthographic processing, that is, the processing of letter identities, location and position within a word, is one of the keys to becoming a skilled reader [15,22,23]. Indeed, several studies show that spatial attention mechanisms are impaired in DD [8,13,24,25], and in pre-reading children that will be future poor readers [8,10,23,26,27]. Spatial attention deficits in DD could be linked to a magnocellular-dorsal (MD) pathway dysfunction [13,14]. The MD pathway originates in the ganglion cells of the retina, passes through the M-layer of the lateral geniculate nucleus, and finally reaches the occipital and parietal cortices, where it plays a key role in motion perception and spatial attention control [13,14]. Children and adults with DD report letter mislocation, increased interference by flanker letters and words (i.e., visual crowding), reduced processing of letter strings, and motion, as well as global scene perception deficits [8,23,26,27].

The theory of DD based on spatial attention mechanism impairments, suggests that an attentional skills training program could produce beneficial effects on reading skills [28]. In particular, in the last few years it has been proposed a training with a specific type of video games, called action video games (AVGs; [6]). The literature shows that AVGs could improve several cognitive skills in healthy adults, such as the speed of processing in terms of response times [29], perception [30], spatial cognition [31] and auditory spatial attention [32], as well as improved multisensory processing in children of 4–5 years treated for 2 weeks [33], probably enhancing the attentional control [34,35].

The beneficial effects of AVGs have also been tested directly on children with DD, showing improvements in reading speed, perceptual and attentional mechanisms [6,8,26,27,36,37,38,39,40], and phonological processing, both in shallow and deep orthographies [37,41]. Moreover, AVG training improved cross-modal attentional shifting [36] also in English-speaking children with DD [37].

The hypotheses at the basis of these effects are linked to the ability of AVGs to improve the functioning of specific neural networks (i.e., fronto-parietal network, prefrontal network, and MD pathway) implicated in both selective spatial attention and reading [26,42]. In a diffusion tensor image study, Gong et al. [43] found significantly strengthened connections in the prefrontal network, limbic system, and sensorimotor network mainly in the right hemisphere of AVG players. In addition, Tanaka et al. [44], comparing gray matter volume in AVG experts and non-experts, using structural magnetic resonance imaging and voxel-based morphometry analysis, revealed significantly larger gray matter volume in the right posterior parietal cortex in AVG experts compared with non-experts.

The speed of transient events and moving objects, the high degree of perceptual and motor load, and the emphasis on peripheral processing of AVGs could improve the functioning of the feedforward stimulus-driven MD pathway [26,36] and the feedback top-down attentional control [42]. The MD pathway is strongly related to reading abilities, indeed several studies have shown a selective deficit in this pathway both in adults and children with DD [45,46,47], underlining that an MD pathway dysfunction—hampering spatial attention—could be one of the multiple causes of DD [26,48].

Thus, it could be supposed that AVG training induces a fronto-parietal plasticity of the top-down prefrontal network of attentional control [42] and the bottom-up parietal network of MD pathway functioning, producing beneficial effects in goal-directed behavior [49,50], such as reading acquisition [51]. In a functional magnetic resonance imaging study, Focker et al. [52] found that the fronto-parietal network was more activated during the processing of visual stimuli in AVG players, especially observed in areas such as frontal pole, the middle frontal gyrus, the postcentral gyrus and the temporo-parietal junction. During the processing of visual stimuli, the connectivity between top-down brain areas and perceptual areas was strengthened in AVG players and this could indicate a signature of higher attentional control. The interaction between top-down and sensory areas appeared mainly regulated by the right temporo-parietal junction and the right middle frontal gyrus, two key areas in mediating more efficient attentional control mechanisms [52]. The reading improvements induced by AVG training in DD could be linked to an enhancement of the efficiency of the fronto-parietal network and MD pathway—controlling the complex interplay between top-down attention and bottom-up spatial processing—that are both critically involved in reading acquisition and consolidation.

Wu and Spence [53] showed the effect of AVGs on top-down attentional control on a visual search task, improving both speed and accuracy. Feature and conjunction visual searches were faster in AVG players, suggesting that video game players developed a better target template to guide search in a top-down manner. In addition, their results suggest that AVGs also improve a top-down guidance of attention to possible target locations, as measured in the dual search task [53].

The effect of AVGs on top-down guidance of attention is extremely useful in a visual search task in which the fundamental problem is the absence of precise advance information about target locations [18]. There are four coordinated stages that perform specific cognitive functions and each of them is by a particular neural signature [18]. The search starts with a preparation stage in which the object or feature to look for is represented in working memory and the prefrontal cortex is involved with top-down attentional control, in particular when no precise spatial information about target locations is available. Preparation stage occurs before the visual display, while the guidance and selection, that are the second and third stages, operate once a search display has been presented. Guidance operates in parallel and globally across the entire visual field independent of the focus of spatial attention, to accumulate information about presence of task-relevant features [18]. A plausible neural basis of spatially global attentional guidance in visual search has been identified in the middle temporal area [54] of the MD pathway. Despite preparation and guidance stages are temporally distinct and functionally dissociable, these are partially functionally linked. The top-down attentional control operates in the preparation stage in a position-independent fashion and could directly drive the spatially feature-based attention during parallel accumulation of information in the guidance stage [18]. The transition from guidance stage to the next, that is the selection stage, is marked by the transition from global analysis across the visual field to a more local and focused attention. Here, the processing resources to candidate target objects at specific locations could be controlled by posterior parietal cortex [55], the frontal eye fields [56], or the thalamus [57]. The final target identification stage that follows object selection is driven by working memory [18]. A recurrent feedback loop between top-down attentional control and spatial attention areas is necessary for integration and discrimination of target features [58].

In addition to previous studies on the effects of AVGs, the aim of this study is to investigate if an AVG training, compared to a non-AVG (NAVG) training in children with DD, improves attentional control measured through a serial conjunction visual search task. In order to have a global picture of the effect of AVGs in children with DD, both pseudoword (i.e., phonological decoding) and word reading abilities have been tested. Furthermore, here we also investigate the cross-modal mapping from visual stimuli to the correspondent spoken words measured by a non-alphanumeric rapid automatized naming (RAN) task.

The hypothesis is that after AVG training children with DD will show improvements in their reading speed [8,36,37] and attentional control both in reaction times (RTs) and accuracy. The improvements on attentional control are hypothesized in RTs because AVGs improve the speed of processing [29] and in accuracy because improving top-down mechanism enhances the feedback loop necessary to complete the visual search more accurately [18,58]. In particular, a reduction of the slope of the distractor set-size—measured as difference between increasing number of distractors—was expected after AVG training. An improvement in the fluency of cross-modal mapping could be also expected because AVGs enhance the cross-sensory attentional shifting ability from visual to auditory stimuli [37].

## 2. Materials and Methods

### 2.1. Participants

Participants were 14 native Italian speaking children (four females and 10 males; mean age = 8.93 years SD = 0.99) with DD. DD was diagnosed based on National Health Service criteria: typical intelligence quotient, no hearing difficulties or neurological deficits, normal or corrected to normal vision [59]. A child received a diagnosis of DD if their speed and/or accuracy in word and pseudoword standardized reading tasks were below −2 standard deviations. The information about video game experience was collected through interviews with parents during a pre-informative briefing about the experimental training. Children with DD did not know the aim of the training and in the previous 6 months did not play AVGs for more than 1 h per week. A crossover intervention study, in which each participant was treated both with AVGs and NAVGs (but two children; *n* = 12) in counterbalanced order, was executed. The study was approved by the Ethics Committee of Psychological Research, University of Padua (Protocol number: 1452; Code: D32B2B803B68E2600F95F0CF66DC42D8).

### 2.2. Reading Tasks

Pseudoword and word texts and lists order administration were counterbalanced between children before and after AVG and NAVG trainings.

#### 2.2.1. Phonological Decoding Tasks

Phonological decoding abilities (speed and number of errors) were measured using three pseudoword texts [60], and three lists of 15 pseudowords each, composed of 2–4 syllables (the same syllables in different order for the three lists; [60]).

#### 2.2.2. Word Reading Tasks

Reading abilities (speed and number of errors) were measured using three word texts (based on [61]), and three lists, of 27 words each, composed of 2–4 syllables [60].

### 2.3. Visual Search Task

**Figure 1:**
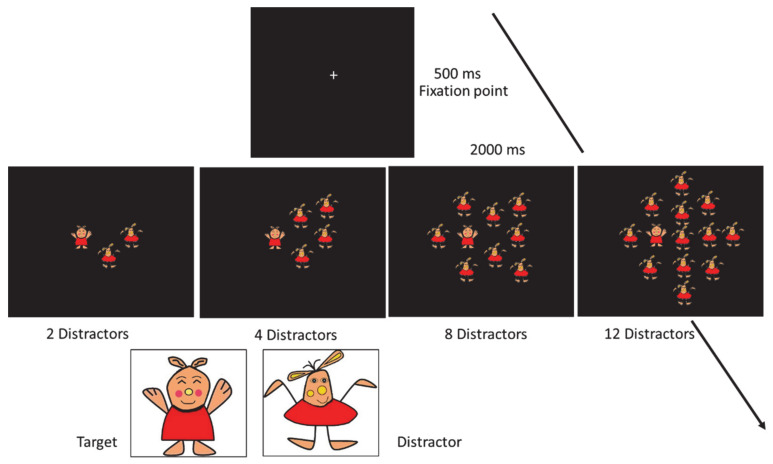
Schematic representation of the serial visual search task with the four possible distractor set-sizes (target present condition is reported). Target and distractor stimuli are also reported.

The experimental procedure and data acquisition were controlled with E-prime 2.0 (Psychology Software, Inc., Sharpsburg, PA, USA). Participants were seated 60 cm away from the screen. The children’ task was to indicate the presence or absence of the target ignoring the distractors with a key press (Y or B on a keyboard, respectively). The stimuli (little puppets) were shown at two eccentricities: at 4.30 deg and 9.07 deg around the center of the screen. The children were asked to keep their eyes on the center of the screen for all duration of each trial. To control that children were fixing at the center of the screen, in eight fixation control trials a stimulus (target or distractor) was shown at the center of the screen. The target and distractors were similar for color, but they differed for the shape. After a small cross (0.1° and 0.6 cd/m^2^), appeared at the center of the screen for 500 msec, target and distractors (both of 2.86° × 3.82°) were shown for 2000 msec. The task was composed of four different set-size conditions (3, 5, 9 and 13 stimuli with or without the target; Figure 1). A total amount of 208 trials were presented (eight fixation control trials; 50 trials for each set-size, 25 trials with target and 25 trials without target).

### 2.4. Non-Alphanumeric RAN Task

The experimental procedure and data acquisition were controlled with E-prime 2.0 (Psychology Software, Inc., Sharpsburg, PA, USA). Cross-modal mapping from visual stimuli to the correspondent spoken words was measured by using a computerized single-item RAN task [62], in which a single-filled colored circle was presented (i.e., red, blue, white and green). Since previous studies showed that alphanumeric RAN tasks are biased by reading experience [63], we used a non-alphanumeric RAN task. Previous studies showed that non-alphanumeric RAN tasks predict later reading performance [64]. Participants were seated 60 cm away from the screen. After a fixation point (500 msec), and a blank screen of 50 msec, a colored circle (diameter = 4.3 deg) appeared at the center of the screen remaining until the children responded. The children’ task was to name the colors of the circles as fast as possible in a microphone connected to a response-box (E-prime 2.0 Psychology Software, Inc., Sharpsburg, PA, USA), which recorded the onset of vocal response. Response’s accuracy was entered by the experimenter, by pressing the corresponding key on the computer keyboard. The inter-trial interval was 1550 msec. A total amount of 32 trials were presented, divided into two blocks of 16 trials each (four trials for each color).

### 2.5. Training Procedure

**Figure 2:**
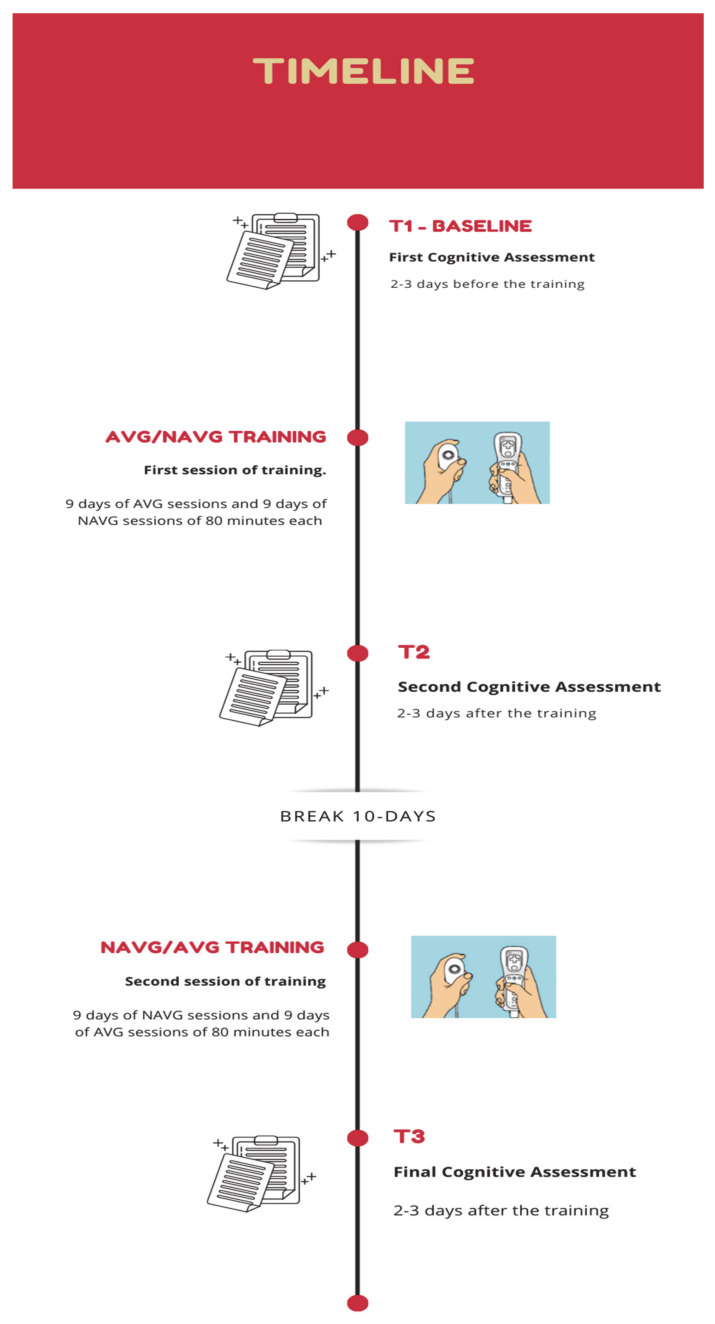
Schematic representation of the timeline of the study.

Participants were individually trained in a dimly lit and quiet room. Participants were tested 2 and 3 days before the start of treatment and re-tested between 2 and 3 days after the end of training. Training consisted of 9 days of AVG sessions and 9 days of NAVG sessions of 80 min each and vice versa. Between T2 and the start of the other training session about 10 days passed. Video games were played standing about 200 cm from a 27-in TV screen. The commercial Wii™ video game and the mini games lists for AVG and NAVG training were the same used in previous research [8,26,36,37]. Similarly to Franceschini and Bertoni [41], the final video game scores of the individual players were recorded after the two training sessions. The timeline of the present study is reported in Figure 2.

## 3. Results

### 3.1. Within-Subject Analysis: Pre vs. Post AVG and Pre vs. Post NAVG

#### 3.1.1. Reading Task

##### Phonological Decoding Tasks

Pseudoword reading speed (syllables per second, syll/sec) improvement was evaluated in AVG and NAVG training session by two separate ANOVAs with 2 times (pre and post training) × 2 tasks (pseudowords lists and pseudowords texts) within-subject design. Results showed a significant main effect of time in the AVG training (F_(1,13)_ = 8.982, *p* = 0.010, η^2^ = 0.409; pre-AVG mean = 0.95 SD = 0.23, post-AVG mean = 1.06 SD = 0.31; mean improvement = 0.11 syll/sec).

In contrast, the main effect of time in the NAVG training was not significant (F_(1,11)_ = 1.558, *p* = 0.238, η^2^ = 0.124; pre-NAVG mean = 1.07 SD = 0.31; post-NAVG mean = 1.11 SD = 0.34; mean improvement = 0.04 syll/sec). Individual data analysis showed that after the AVG training session, 8 out of 14 players (about 60%) improved their pseudoword reading speed more than the mean improvement after the NAVG training session.

The same ANOVAs considering the number of errors as dependent variable were not significant neither after AVG nor after NAVG training session (AVG: F_(1,13)_ = 0.188, *p* = 0.67, η^2^ = 0.014; pre-AVG mean = 7.57 SD = 5.16, post-AVG mean = 7.93 SD = 3.85; mean improvement = −0.36 errors; NAVG: F_(1,11)_ = 0.059, *p* = 0.813, η^2^ = 0.005; pre-NAVG mean = 8.50 SD = 3.86, post-NAVG mean = 8.71 SD = 5.36; mean improvement = −0.21 errors).

Thus, the reading improvements after the AVG training were characterized by increased phonological decoding speed without any cost in accuracy, confirming the previous experimental evidence [6,8,36,37,41].

##### Word Reading Tasks

Word reading speed (syll/sec) improvement was evaluated in AVG and NAVG training by two separate ANOVAs with 2 times (pre and post training) × 2 tasks (word lists and word texts) within-subject design. Results did not show any significant effect neither after AVG nor after NAVG training session (AVG: F_(1,13)_ = 0.084, *p* = 0.776, η^2^ = 0.006; pre-AVG mean = 1.25 SD = 0.40, post-AVG mean = 1.27 SD = 0.49; mean improvement = 0.02 syll/sec; NAVG: F_(1,11)_ = 0.062, *p* = 0.807, η^2^ = 0.006; pre-NAVG mean = 1.30 SD = 0.44, post-NAVG mean = 1.27 SD = 0.41; mean improvement = −0.03 syll/sec). The same ANOVAs considering the number of errors as dependent variable, did not show any significant effect neither after AVG nor after NAVG training session (AVG: F_(1,13)_ = 0.006, *p* = 0.941, η^2^ = 0.001; pre-AVG mean = 12.14 SD = 8.14, post-AVG mean = 12.25 SD = 6.39; mean improvement = −0.11 errors; NAVG: F_(1,11)_ = 0.989, *p* = 0.341, η^2^ = 0.082; pre-NAVG mean = 11.75 SD = 6.08, post-NAVG mean = 13.38 SD = 8.99; mean improvement = −1.63 errors).

#### 3.1.2. Visual Search Task

The RTs (in msec) and accuracy (in rate) in the visual search task were analyzed by using two ANOVAs with a 2 times (pre and post training) × 2 task conditions (target present and target absent) × 4 set-sizes (2, 4, 8 and 12 distractors) within-subject design for each training (AVG and NAVG).

##### RTs

**Figure 3:**
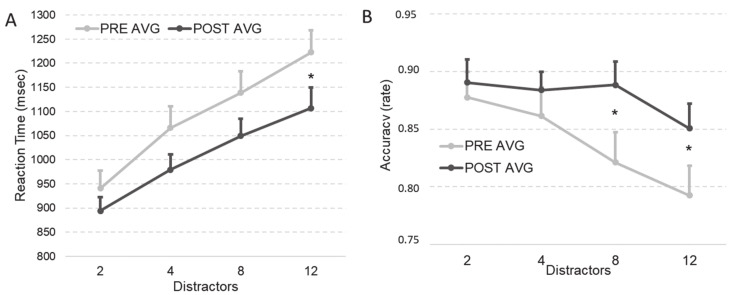
Panel (**A**): reaction times (in msec) in visual search before (PRE AVG) and after (POST AVG) action video game training. Panel (**B**): accuracy (in rate) in visual search before (PRE AVG) and after (POST AVG) action video game training. Error bars report the mean standard error. The asterisks indicate the significant differences.

In the ANOVA for the AVG training, the main effects of task condition (F_(1,13)_ = 27.16, *p* < 0.001, η^2^ = 0.676), and set-size (F_(1,13)_ = 138.11, *p* < 0.001, η^2^ = 0.914) were significant. In addition, the task condition × set-size interaction (F_(1,13)_ = 45.36, *p* < 0.001, η^2^ = 0.777) was significant. Crucially for our hypothesis, time × set-size interaction was also significant (F_(1,13)_ = 5.56, *p* = 0.035, η^2^ = 0.30). Planned comparisons showed that the RTs reduction was present in the more difficult set-size condition (12 distractors: *t*_(13)_ = 2.192, *p* = 0.047; pre-AVG mean = 1222 SD = 170; post-AVG mean = 1106 SD = 157; see Figure 3, Panel A) and not in the other set-size conditions (eight distractors: *t*_(13)_ = 2.066, *p* = 0.059; pre-AVG mean = 1138 SD = 168; post-AVG mean = 1049 SD = 130; four distractors: *t*_(13)_ = 1.705, *p* = 0.112; pre-AVG mean = 1066 SD = 164; post-AVG mean = 979 SD = 118; two distractors: *t*_(13)_ = 1.158, *p* = 0.268; pre-AVG mean = 940 SD = 134; post-AVG mean = 894 SD = 106). Planned comparison showed that the AVG training reduced the slope of the set-size effect measured as the RTs difference between the smaller (i.e., two distractors) and the larger (i.e., 12 distractors) set-size conditions (*t*_(13)_ = 2.307, *p* = 0.038; pre-AVG slope: mean = 281 SD = 89; post-AVG slope: mean = 212 SD = 91).

In the ANOVA for the NAVG training, the main effects of task condition (F_(1,11)_ = 25.62, *p* < 0.001, η^2^ = 0.70) and set-size (F_(1,11)_ = 118.99, *p* < 0.001, η^2^ = 0.915) were significant. In addition, the task condition × display size interaction (F_(1,11)_ = 27.65, *p* < 0.001, η^2^ = 0.715) was significant. Importantly, time × set-size interaction was not significant (F_(1,11)_ = 0.254, *p* = 0.62, η^2^ = 0.023).

##### Accuracy

In the ANOVA for the AVG training main effects of time (F_(1,13)_ = 7.25, *p* = 0.018, η^2^ = 0.358) and set-size were significant (F_(1,13)_ = 9.75, *p* = 0.008, η^2^ = 0.429). In addition, the task condition × set-size interaction was significant (F_(1,13)_ = 11.07, *p* = 0.005, η^2^ = 0.460). Crucially for our hypothesis, time × set-size interaction was also significant (F_(1,13)_ = 4.68, *p* = 0.048, η^2^ = 0.265). Planned comparisons showed that the accuracy improvement was present in the more difficult set-size conditions (12 distractors: *t*_(13)_ = 2.877, *p* = 0.013; pre-AVG mean = 0.79 SD = 0.09; post-AVG mean = 0.85 SD = 0.08; eight distractors: *t*_(13)_ = 3.312, *p* = 0.006; pre-AVG mean = 0.82 SD = 0.09; post-AVG mean = 0.88 SD = 0.07 see Figure 3, Panel B), but not in the other set-size conditions (four distractors: t_(13)_ = 1.006, *p* = 0.33; pre-AVG mean = 0.86 SD = 0.09; post-AVG mean = 0.88 SD = 0.06; two distractors: *t*_(13)_ = 0.762, *p* = 0.46; pre-AVG mean = 0.88 SD = 0.08; post-AVG mean = 0.89 SD = 0.07). Moreover, planned comparison showed that the AVG training nullified the slope of the set-size effect measured as the accuracy difference between the smaller (i.e., two distractors) and the larger (i.e., 12 distractors) set-size conditions (pre-AVG: *t*_(13)_ = 3.941, *p* = 0.002; two distractors: mean = 0.87 SD = 0.08; 12 distractors: mean = 0.79 SD = 0.09; post-AVG: *t*_(13)_ = 1.418, *p* = 0.18; two distractors: mean = 0.89 SD = 0.07; 12 distractors: mean = 0.85 SD= 0.08; one-tail *t*-test pre- vs. post-AVG slope: (*t*_(13)_ = 1.879, *p* = 0.04; pre-AVG slope: mean = 0.08 SD = 0.08; post-AVG slope: mean = 0.04 SD = 0.11).

In the ANOVA of accuracy in the NAVG training only the main effect of the set-size was significant (F_(1,11)_ = 13.99, *p* = 0.003, η^2^ = 0.56). Importantly, time × set-size interaction was not significant (F_(1,11)_ = 3.07, *p* = 0.11, η^2^ = 0.218).

#### 3.1.3. Non-Alphanumeric RAN Task

The vocal RTs (in msec) in the RAN task were analyzed by two ANOVAs with two times (before and after) within-subject design for each training (AVG and NAVG). Neither ANOVA on AVG nor NAVG training showed any significant effect (AVG: F_(1,13)_ = 0.10, *p* = 0.757, η^2^ = 0.008; pre-AVG mean = 876 SD = 245, post-AVG mean = 848 SD = 232; mean improvement = 28 msec; NAVG: F_(1,11)_ = 1.364, *p* = 0.267 η^2^ = 0.11; pre-NAVG mean = 870 SD = 234, post-NAVG mean = 796 SD = 172; mean improvement = 74 msec).

#### 3.1.4. Action Video Game Ability after Training

Similarly to Franceschini and Bertoni [41], we recorded the video game scores of participants in order to control the players’ games efficiency after AVG training. The median game score was calculated after AVG training. We divided the sample in two sub-groups: the children with a game score greater than the median score (high score players, HSPs, *n* = 7; three females and four males) and those who showed a game score lower than the median score (low score players, LSPs, *n* = 7: one female and six males).

The change of pseudoword reading speed (syll/sec) between pre- and post-AVG training was analyzed with two non-parametric Wilcoxon tests for HSP and LSP sub-groups. The results show an improvement in pseudoword reading speed only in HSP sub-group (Z = −2.20, *p* = 0.028; pre-AVG mean = 1.06 SD = 0.23, post-AVG mean = 1.23 SD = 0.34; phonological decoding speed improvement = 0.17 syll/sec SD = 0.14), but not in LSP sub-group (Z = −1.37, *p* = 0.17; pre-AVG mean = 0.85 SD = 0.19, post-AVG mean = 0.89 SD = 0.15; phonological decoding speed improvement = 0.04 SD = 0.07).

The non-parametric Wilcoxon tests in HSP and LSP sub-groups were also conducted to test the possible difference in RTs and accuracy of the set-size slope, indexed as the difference between the smaller (i.e., two distractors) and the larger (i.e., 12 distractors) set-size conditions. The RTs and accuracy set-size slopes were reduced only in HSP sub-groups (RTs: Z = −2.20 *p* = 0.028, pre-AVG mean = 278 SD = 79, post-AVG mean = 175 SD = 57; accuracy: Z = −2.20 *p* = 0.028, pre-AVG mean = 0.08 SD = 0.08, post-AVG mean = −0.2 SD = 0.08), showing an attentional control enhancement only after an efficient AVG training indexed by higher video game score.

### 3.2. Between-Subjects Analysis

These findings demonstrate specific improvements in phonological decoding speed and attentional control selectively induced through efficient AVG training.

However, direct comparisons between the two trainings and between HSP and LSP after AVG training are necessary to stringently demonstrate the selective effects of AVG and HSPs after AVG training on phonological decoding speed and attentional control indexed by RTs and accuracy of the set-size slope.

The ideal ANOVA with two groups (AVG and NAVG) by three times (T1, T2 and T3) design cannot be carried-out, because 2 out of 14 (about 15%) of participants were trained only with AVGs between T1 and T2.

#### 3.2.1. T1 vs. T2

Similarly to the typical between-subjects intervention studies e.g., [8,27,36,37], in this first between-subjects analysis, we directly compared the improvements (i.e., post-training–pre-training performance) induced by the AVG and NAVG training in our two groups of children with DD (*n* = 8 and *n* = 6, respectively). This first analysis allows us a partial comparison between AVG and NAVG training in children with DD without any previous AVG experience.

To investigate the selective clinical effect of the AVG treatment on reading skills, we also compared the improvements induced by the AVG and NAVG training vs. one-year (8760 h) of spontaneous development of the phonological decoding speed [65]. If the AVG training actually has a robust clinical effect on phonological decoding speed, then we should find a significant difference in the NAVG control group, but not in the AVG group, indicating that 12 h of AVGs accelerate pseudoword reading similarly to 1 year of spontaneous development.

##### Phonological Decoding Tasks

Independent-samples *t*-test (one-tail) showed that the pseudoword reading speed improvement (syll/sec) was significantly different between AVG and NAVG groups (*t*_(12)_ = 1.92, *p* = 0.04; mean AVG improvement between T1 and T2 = 0.12 syll/sec SD = 0.15 vs. mean NAVG improvement between T1 and T2 = −0.005 syll/sec SD = 0.07).

In addition, one-sampled *t*-test showed that the reading speed improvement in AVG group was not significantly different (*t*_(7)_ = −0.59, *p* = 0.29) to the spontaneous reading development (i.e., 0.15 syll/sec [65]).

Independent-samples *t*-test (one-tail) showed that the pseudoword reading errors improvement was not significantly different between AVG and NAVG group (*t*_(12)_ = −0.24, *p* = 0.40; mean AVG improvement between T1 and T2 = −0.31 SD = 2.99 vs. mean NAVG improvement between T1 and T2 = 0.08 SD = 3.02).

##### Visual Search Task

Independent-samples *t*-test (one-tail) on RTs slope (RTs difference between 12 and 2 set-size in msec) reduction between AVG and NAVG group was marginally significant (*t*_(12)_ = 1.47, *p* = 0.08; mean AVG slope improvement between T1 and T2 = 80 msec SD = 143 vs. mean NAVG slope improvement between T1 and T2 = −33 msec SD = 140).

Independent-samples *t*-test (one-tail) on accuracy slope (RTs difference between 2 and 12 set-size in rate) reduction between AVG and NAVG group was not significant (*t*_(12)_ = 1.07, *p* = 0.15; mean AVG slope improvement between T1 and T2 = −0.01 SD = 0.09, mean NAVG slope improvement between T1 and T2 = −0.06 SD = 0.06).

#### 3.2.2. T2 vs. T3

In the second between-subjects analysis, we compared the improvements induced by the AVG and NAVG training between T2 and T3 in our two groups (now *n* = 6 for both groups). This type of comparison is not the typical analysis of the between-subjects intervention studies [41,42], because in this case the NAVG control group now had previous AVG experience. Indeed, it cannot be excluded that the effect of the previous AVG training is “carried over” to the following NAVG training. Thus, this comparison should be considered with caution.

##### Phonological Decoding Tasks

Independent-samples *t*-test (one-tail) on reading speed (syll/sec) showed no significant difference between AVG and NAVG (*t*_(10)_ = 0.39, *p* = 0.35; mean AVG improvement between T2 and T3 = 0.07 SD = 0.09, mean NAVG improvement between T2 and T3 = 0.11 SD = 0.17).

The one-sample *t*-test (one-tail) showed that the reading speed improvement in the AVG group was marginally different to the spontaneous reading development (*t*_(5)_ = −2.003, *p* = 0.051; mean AVG improvement between T2 and T3 = 0.07 SD = 0.09).

Independent-samples *t*-test (one-tail) on reading errors showed no significant difference between AVG and NAVG (*t*_(10)_ = −0.04, *p* = 0.48; mean AVG improvement between T2 and T3 = −0.42 SD = 3.50, mean NAVG improvement between T2 and T3 = −0.50 SD = 3.18).

##### Visual Search Task

Independent-samples *t*-test (one-tail) on RTs showed no significant difference in the reduction of the slope between AVG and NAVG (*t*_(10)_ = 0.44, *p* = 0.33; mean AVG improvement between T2 and T3 = 55 SD = 59, mean NAVG improvement between T2 and T3 = 78 SD = 114).

Independent-samples *t*-test (one-tail) on accuracy showed no significant difference in the reduction of the slope between AVG and NAVG (*t*_(10)_ = 1.23, *p* = 0.13; mean AVG improvement between T2 and T3 = −0.09 SD = 0.07, mean NAVG improvement between T2 and T3 = −0.03 SD = 0.11).

#### 3.2.3. Comparison between First NAVG Group vs. Second NAVG Group

In the third between-group analysis, we tested the effect of previous AVG training on the next NAVG training directly comparing the improvements in the first NAVG group (between T1 and T2; *n* = 6, without previous AVG experience) and the second NAVG group (between T2 and T3; *n* = 6, with previous AVG training experience).

To investigate the clinical effect of the previous AVG training on reading skills, we also compared the improvements induced by the first and second NAVG group vs. one-year of spontaneous development of the phonological decoding speed. If the previous AVG training actually has a robust clinical effect on the subsequent NAVG training, then we should find a difference only in the first NAVG control group.

##### Reading Task: Phonological Decoding Tasks

Independent-samples *t*-test (one-tail) on reading speed (syll/sec) showed a marginal difference between NAVG groups (*t*_(10)_ = 1.44, *p* = 0.09; mean NAVG improvement between T1 and T2 = −0.005 SD = 0.07, mean NAVG improvement between T2 and T3 = 0.11 SD = 0.17).

One-sample *t*-test (one-tail) showed that the first NAVG group is significantly different to the spontaneous reading development (*t*_(5)_ = −5.62, *p* = 0.001; mean NAVG improvement between T1 and T2 = −0.005 SD = 0.07), whereas the second NAVG group was not significantly different to 0.15 syll/sec (*t*_(5)_ = −0.63, *p* = 0.28; mean NAVG improvement between T2 and T3 = 0.11 SD = 0.17).

Independent-samples *t*-test (one-tail) on reading errors showed no significant difference between NAVG groups (*t*_(10)_ = −0.33, *p* = 0.38; mean NAVG improvement between T1 and T2 = 0.08 SD = 3.02, mean NAVG improvement between T2 and T3 = −0.5 SD = 3.18).

##### Visual Search Task

Independent-samples *t*-test (one-tail) on RTs showed a marginal difference in the reduction of the slope between two NAVG groups (*t*_(10)_ = 1.50, *p* = 0.08; mean NAVG improvement between T1 and T2 = −33 SD = 140, mean NAVG improvement between T2 and T3 = 78 SD = 114).

Independent-samples *t*-test (one-tail) on accuracy showed no significant difference in the reduction of the slope between two NAVG groups (*t*_(10)_ = 0.62, *p* = 0.28; mean NAVG improvement between T1 and T2 = −0.06 SD = 0.06), mean NAVG improvement between T2 and T3 = −0.03 SD = 0.11).

#### 3.2.4. Comparison between Total AVG and First NAVG Groups

Once confirmed the plausibility of the effect of the previous AVG training on the subsequent NAVG training, to better investigate the specific role of AVG training on phonological decoding speed and attentional control, in the fourth between-subjects analysis, we compared all 14 children with DD treated with the AVG training (i.e., the first AVG group between T1 and T2 and the second AVG group between T2 and T3) vs. the first NAVG control group.

##### Reading Task: Phonological Decoding Tasks

One-sample *t*-test (one-tail) on reading speed (syll/sec) showed a significant difference between AVG and first NAVG group (*t*_(13)_ = 3.15, *p* = 0.004; mean AVG improvement between pre and post training = 0.10 SD = 0.12, mean NAVG improvement between T1 and T2 = −0.005).

One-sample *t*-test (one-tail) on reading errors showed no significant difference between AVG and first NAVG group (*t*_(13)_ = −0.53, *p* = 0.31; mean AVG improvement between pre and post training = −0.36 SD = 3.08, mean NAVG improvement between T1 and T2 = 0.08).

##### Visual Search Task

One-sample *t*-test (one-tail) on RTs showed significant difference in the reduction of the slope between AVG and first NAVG group (*t*_(13)_ = 3.40, *p* = 0.002; mean AVG improvement between pre and post training = 69 SD = 112, mean NAVG improvement between T1 and T2 = −33).

One-sample *t*-test (one-tail) on accuracy showed significant difference in the reduction of the slope between AVG and first NAVG group (*t*_(13)_ = 3.96, *p* = 0.001; mean AVG improvement between pre and post training = 0.05 SD = 0.09, mean NAVG improvement between T1 and T2 = −0.05).

##### Comparison between HSP and LSP Groups

Finally, to stringently test the effects of AVG training efficiency, in our fifth between-subjects analysis, we directly compared HSPs and LSPs after the AVG training.

##### Reading Task: Phonological Decoding Tasks

Independent-samples *t*-test (one-tail) on reading speed (syll/sec) showed a significant difference between HSP and LSP AVG group (*t*_(12)_ = 1.99, *p* = 0.035; mean HSP improvement between pre and post training = 0.17 SD = 0.14, mean LSP pre and post training = 0.04 SD = 0.07).

##### Visual Search Task

Independent-samples *t*-test (one-tail) on RTs showed no significant difference in the reduction of the slope between HSP and LSP AVG group (*t*_(12)_ = 1.13, *p* = 0.14; mean HSP improvement between pre and post training = 103 SD = 89, mean LSP improvement between pre and post training = 36 SD = 129).

Independent-samples *t*-test (one-tail) on accuracy showed significant difference in the reduction of the slope between HSP and LSP AVG group (*t*_(12)_ = 2.13, *p* = 0.028; mean HSP improvement between pre and post training = 0.09 SD = 0.07, mean LSP improvement between pre and post training = −0.001 SD = 0.09).

## 4. Discussions

In this partial crossover intervention study, we investigated the effects of a visuo-attentional training based on AVGs in children with DD. The results show that 12 h of AVG training improve pseudoword reading speed and attentional control in a serial visual search task. These results are confirmed not only using two independent comparisons within each training session (i.e., pre vs. post training within-subject analysis), but also when more stringent between group comparisons (i.e., AVG vs. NAVG between-subject analysis) on improvements were executed. In contrast, there are no beneficial effects of AVGs in word reading performance and in cross-modal mapping measured through non-alphanumeric RAN.

The improvement in pseudoword phonological decoding speed is in line with the literature [8,36,37,41]. The pseudoword reading skills is based on sub-lexical mechanisms that are driven not only by the bottom-up MD pathway [45], but also by prefrontal top-down attention [42].

It is demonstrated that the intrinsic characteristics of AVGs, such as the speed in terms of transient events and moving objects, the high degree of perceptual and motor load, and the emphasis on peripheral processing, enhance the “action” stream that include both the MD pathway [26] and prefrontal top-down attention [42].

AVG play enhances various aspects of attentional control now better understood as changes in the capacity to rapidly shift between a distributed versus a focused attentional state in the spatial resolution [42], necessary in both pseudoword reading [36] and visual search [18].

On the contrary, the absence of word reading skills improvement could be linked to an absence of enhancement of occipito-temporal ventral functioning also demonstrated with no vocal RTs amelioration in the cross-modal mapping of non-alphanumeric RAN task. These results do not replicate the effects observed by Łuniewska and colleagues [39] in their comparison between AVGs and phonological/phonic training, where practice effects were observed in RAN tasks as well as in multiple cognitive tasks. The absence of an effect in non-alphanumeric RAN task seems to exclude that the observed beneficial effects in phonological decoding and attentional control could be due to a generalized faster speed of processing.

The main result is the enhancement of attentional control efficiency (RTs and accuracy) in the serial visual search task. In particular, these results showed a reduction of the slope in the RTs and a flattening of the slope in the accuracy, in which AVG training decreases and nullifies the effect of the distractor set-size, respectively.

The attentional control in conjunction visual search is the result of a combination of different and coordinated neurocognitive stages, in which preparation, guidance, selection and identification follow each other [18]. The improvement in top-down attentional control appears to be disproportionately enhanced after playing AVGs as compared to stimulus-driven attention [42]. Attentional control abilities are regulated by a constant interplay between previously characterized bottom-up and top-down attentional networks [50,66]. The different effects of AVGs on these two mechanisms may potentially reflect the fact that bottom-up attention mechanisms are simply less plastic, and that top-down attentional control, by calling upon cognitive flexibility, working memory and some forms of inhibitory control, is likely highly plastic [42,67].

AVGs are characterized by three key elements that must be present in an interactive environment to enhance attentional control: (1) fast pacing, or the need for making decisions under time pressure relative to players’ abilities [42]; (2) the need to filter distractors and sustain global attention across the entire game environment for a significant period of time, and; (3) the need to switch between modes of processing, such as the many switches from a global attentional control and a more local and focused attentional state as a function of the ever-changing game contingencies [66].

The second and third key elements of AVGs are well linked to the second and third neurocognitive stages of attentional control, i.e., guidance and selection. The performance improvements in our visual search task could be linked to an enhancement in the central stages of visual search. Indeed, the guidance stage operates for a parallel accumulation of information globally across the entire visual field, being guided by MD pathway [14] or initial feedforward hierarchy underlying the implicit processing for “vision at a glance” [66]. Evidence regarding the improvement in the distributed attentional control after AVG training in children with DD is yet been demonstrated in Franceschini et al. [27], in which the results showed a better global processing, and in Franceschini et al. [36] in which distributed attention was ameliorated in the probe condition of a single report visual attention span task.

The attentional control improvements recorded in our sample of children with DD could be linked also to the selection stage of visual search in which a local analysis of the visual field guided by later “vision with scrutiny”, in which reverse hierarchy routines focus attention to specific, active and low-level units [24,68]. Evidence about the improvement in the focused attention after AVG training in children with DD has already been demonstrated in Franceschini and colleagues [36,37] in which the results showed an enhancement in the cue condition of a single report visual attention span task, both in Italian [36] and English-speaking children [37]. The enhancement of visual search performances after AVGs linked to both guidance and selection stages could be also congruent with the reduction of crowding effect after AVG training in children with DD [8,69].

The AVG training could enhance the fluidity switch between distributed and focalized attentional states, both important for an efficient top-down attentional control [42].

The improvement in both global and local attentional control is also suggested by the pseudoword reading speed improvements. Indeed, the pseudoword reading requires more attentional resources than word reading [23], with an initial global attentional control sustained by MD pathway (i.e., guidance stage in visual search), and then a more focused attention on specific graphemes, sustained by posterior parietal cortex (i.e., selection stage in visual search; [70]).

Another possible interpretation of the attentional control improvement measured in the visual search task could be linked to an earlier attentional selection enhancement. In this way, an efficient signal-to-noise exclusion mechanism with a better filtering between target and distractors could explain the enhancement in visual search. This interpretation could be congruent with relevant hypothesis of DD such as: (i) the multisensory “sluggish attentional shifting” in DD [71]; (ii) the “perceptual noise exclusion” deficit in DD [72,73] and a general “neural noise” enhancement in DD [74].

Reading speed, but not reading accuracy, is improved after AVGs. These results could suggest that to obtain a general reading enhancement it would be necessary to combine traditional phonological and orthographic treatments that work on accuracy with visuo-attentional trainings that enhance the speed of processing and on general-domain cognitive skills, such as attentional control and that caused beneficial cascade effects on reading speed.

It has been shown that the beneficial effects of the AVG training should be related to the improvement that could be considered as an index of the engagement of children in treatment and of plasticity of their attentional control [41]. Here we show that DD children with higher video game scores, after AVG training, improved both in attentional control and pseudoword phonological decoding speed. In addition, direct comparisons between the two groups strongly confirm the specific role of an efficient AVG training in reading speed and attentional control improvement in children with DD. The clinical relevance of the result found in pseudoword decoding speed can be appreciated by noting that HSPs—after 12 h of efficient AVGs—obtained a phonological decoding improvement of 0.17 syll/sec, higher than the mean improvements expected in an Italian child with DD (0.15 syll/sec) after 1 year of spontaneous reading development [65]. Furthermore, improvements in pseudoword reading speed obtained after AVG training were bigger than those obtained by the highly demanding traditional phonological and orthographic treatments, that are equal to the letter-to-speech integration training [75].

Pseudoword phonological decoding, bottom-up (global) and top-down (focused) attentional control enhancements in children with DD after AVG training, sustain the right fronto-parietal network deficit theory for DD [37,76,77,78]. Accordingly, a single pulse transcranial magnetic stimulation delivered on the right frontal eye fields of the dorsal fronto-parietal network was able to interfere with both global (“vision at a glance” or guidance stage) and focused (“vision with scrutiny” or selection stage) attentional control mechanisms [79]. However, to better understand functional and structural connectivity of the fronto-parietal networks in people with DD, future neuroimaging and psychophysiological studies are needed. Recently, it has been demonstrating that stronger connectivity between dorsal fronto-parietal network regions is associated with faster evidence accumulation and speeded perceptual decisions [80]. Thus, our results in attentional control allow us to hypothesize a right dorsal fronto-parietal dysfunction in people with DD. Accordingly, a recent review and meta-analysis of magnetic resonance imaging studies have localized in the right parietal lobe, possible brain differences associated with DD risk in children before reading instruction that might not be shaped by language experiences during the first years of life [81].

Finally, our comparisons between the first and second NAVG group suggest that these right fronto-parietal attentional mechanisms—underlying reading difficulties in children with DD—once stimulated and unlocked by the efficient attentional training could not stop immediately after the end of AVG training. Although this potential effect should be confirmed by further studies and may complicate the interpretation of the results underlying the cross-over design, it could be remarkably challenging from a preventional perspective. Indeed, a brief experience with AVGs could induce effects that continue even after the end of this stimulation in the following experiences, potentially opening the way to short preventive programs for long-lasting effects on future biobehavioral development [33].

## 5. Conclusions

This preliminary study shows that AVG training appears to improve different stages of attentional control in children with DD. In particular, both bottom-up distributed and top-down focused attention could be enhanced. Thus, AVG training could stimulate the MD pathway and the dorsal fronto-parietal network, which are both involved in phonological decoding as well as in serial visual search. The significance of these results is that an efficient visuo-attentional training can simultaneously enhance both attentional mechanisms (i.e., global-distributed and local-focused) required during pseudoword phonological decoding (i.e., parallel letter-string processing and graphemic parsing).

## Data Availability

The datasets analysed during the current study are available from the corresponding author on reasonable request.
